# Cortical Activation during Swallowing Exercise Tasks: an fNIRS Pilot Study

**DOI:** 10.1007/s00455-024-10730-1

**Published:** 2024-07-09

**Authors:** Denise Mae N. Chua, Karen Man-Kei Chan

**Affiliations:** Swallowing Research Laboratory, Faculty of Education, The University of Hong Kong, 7/F, Meng Wah Complex, Pokfulam, Hong Kong

## Abstract

This pilot study used functional near-infrared spectroscopy (fNIRS) to examine brain activity in selected regions of the left motor and sensory cortex while doing swallowing-related tasks. Specifically, differences in cortical activation during normal saliva swallows, effortful swallows, and tongue pressing were investigated. Nine healthy, right-handed adults (5 female, 4 male; Age: 22–30 years) were recruited. The tasks included were (1) normal saliva swallowing, (2) effortful saliva swallowing, and (3) lingual pressing against the palate. Each task was completed three times in a block, for a total of five blocks. Blocks were randomized and presented with set time intervals using PsychoPy. Motor activity was highest during effortful swallows, followed by normal swallows, and lingual presses. Activation in the sensory region was not significantly different across tasks; however, effortful swallows elicited the highest mean peak activation. Our findings suggest that fNIRS can be a viable imaging method used to examine differences in cortical activity in the context of swallowing. Its applicability in future dysphagia research should be explored.

## Introduction

The act of swallowing requires complex neurological coordination and any damage to relevant cortical networks could compromise swallow safety and efficiency [1]. In healthy adults, multiple studies have confirmed the significant involvement of the primary motor and sensory cortices in swallowing [1–5], and lesions to these sites can cause motor or sensory swallowing deficits [6]. With this, it is not surprising that the diagnosis of neurological conditions such as stroke, Parkinson’s Disease, or dementia are heavily associated with the development of dysphagia or swallowing problems [7].

Dysphagia affects thousands of people globally and speech therapists have routinely prescribed swallowing exercises to rehabilitate swallowing functions [8]. Specifically, tongue-strengthening and effortful swallow exercises are two common rehabilitative exercises that effectively improve swallowing-related physiology [9, 10]. Apart from investigating the behavioral outcomes of these exercises, it is equally important to understand how these exercises address the neurological causes of dysphagia. Despite their widespread use, the direct effects of swallowing-related exercises on brain activity are limited. Tongue-strengthening or lingual pressing exercises have been found to induce plasticity in the motor cortex [11]. In a case presented by Malandraki et al. (2011) [12], neural activation in a dysphagic post-stroke patient increased significantly after eight weeks of lingual training. Similar findings cannot be concluded for other dysphagia rehabilitative techniques such as the effortful swallow exercise. Compared to normal saliva swallows, effortful swallows have been found to elicit more activation in both the motor and sensory cortices [13], and it can be hypothesized that effortful swallow training can also promote neuroplastic changes. Additionally, doing effortful swallows recruit a variety of oral, pharyngeal, and esophageal muscles [9]; thus, could potentially induce stronger and more neural activation as compared to tongue-strengthening exercises.

Existing neuroimaging studies in the field have mostly focused on identifying brain regions relevant to normal swallowing using functional magnetic resonance imagining (fMRI) [1–5]. In terms of swallowing maneuvers, Peck et al. (2010) [13] investigated neural activity of healthy adults during dry saliva swallowing, effortful swallowing, and the Medelsohn maneuver. Ogura et al. (2012) [14], on the other hand, measured brain activity during oral exercises tasks such as lip-pursing, lip-stretching, tongue protrusion, tongue lateralization, and oral ball-rolling. Increased activation in multiple regions of the brain, particularly in the precentral motor and postcentral sensory regions, was observed in both studies. Tasks the required more muscle movement and coordination (i.e., effortful swallow, Mendelsohn maneuver, oral ball-rolling) elicited higher cortical activation as opposed to simpler tasks (i.e., dry saliva swallowing, isolated lip and tongue movements). However, a knowledge gap on the potential rehabilitation effects of these techniques on specific population groups (i.e., older adults, dysphagic patients) remain unexplored likely due to limitations with fMRI data acquisition. Doing swallowing-related tasks inside an MRI machine is not naturalistic and may not be medically possible. Additionally, monitoring and ensuring task accuracy can become challenging. It is worthwhile to pursue the use of other brain imaging modalities that can address these constraints to further examine swallowing-related brain activity.

In recent years, the use of a more portable and non-invasive neuroimaging technique called functional near-infrared spectroscopy (fNIRS) in the field of swallowing has been emerging [15]. Cortical activity reflected by hemodynamic concentration in specific regions of the brain are measured using near infrared light-emitting optodes placed on the scalp. Data collection using fNIRS can be done in an upright sitting position, allowing us to collect swallowing-related information in a naturalistic posture. As compared to fMRI, fNIRS is also more tolerable to head movements [16] making it more feasible to collect data while doing exercise tasks such as lingual pressing. As of writing, published studies have investigated swallowing-related brain activity in relation to different taste stimuli [17], the use of laryngeal vibration [18], and the use of motor imagery [19, 20]. To our knowledge, no study has examined and compared neural activation during tongue-strengthening and effortful swallowing exercises using fNIRS. We aim for this study to help advance the use of fNIRS in the field and most importantly, contribute to the current knowledge gap of how these exercises induce and promote swallowing-related neuroplasticity.

## Objective of the Study

This study examines hemodynamic activity in selected regions of the primary motor and sensory cortex while doing swallowing-related tasks using fNIRS. Differences in activation during normal saliva swallows, effortful swallows, and lingual pressing was investigated. We hypothesized that all three tasks will elicit greater activation in the motor region as compared to the sensory region due to various muscle recruitment and activation. Additionally, we expect that effortful swallowing will lead to greater activity in both regions as compared to normal swallowing and lingual presses.

## Methods

### Participants

Ethical approval was granted by the university’s review board before the commencement of data collection. A total of nine participants aged 22 to 30 (Male = 4; Female = 5) were recruited for this study. All participants were dominantly right handed as assessed by the Edinburgh Handedness Inventory – Short Form [21]. Based on self-report, none of the participants had a history of swallowing problems or had diseases that were associated with swallowing problems (i.e., brain injury, stroke, head and neck cancer). Apart from wisdom tooth extraction, none had received surgical intervention to the ear, nose, throat, or brain region. Rationale and procedures of the study were thoroughly explained before the experiment and written informed consent was obtained. No compensation was provided for participation in this study.

### fNIRS Measurements

**Fig. 1:**
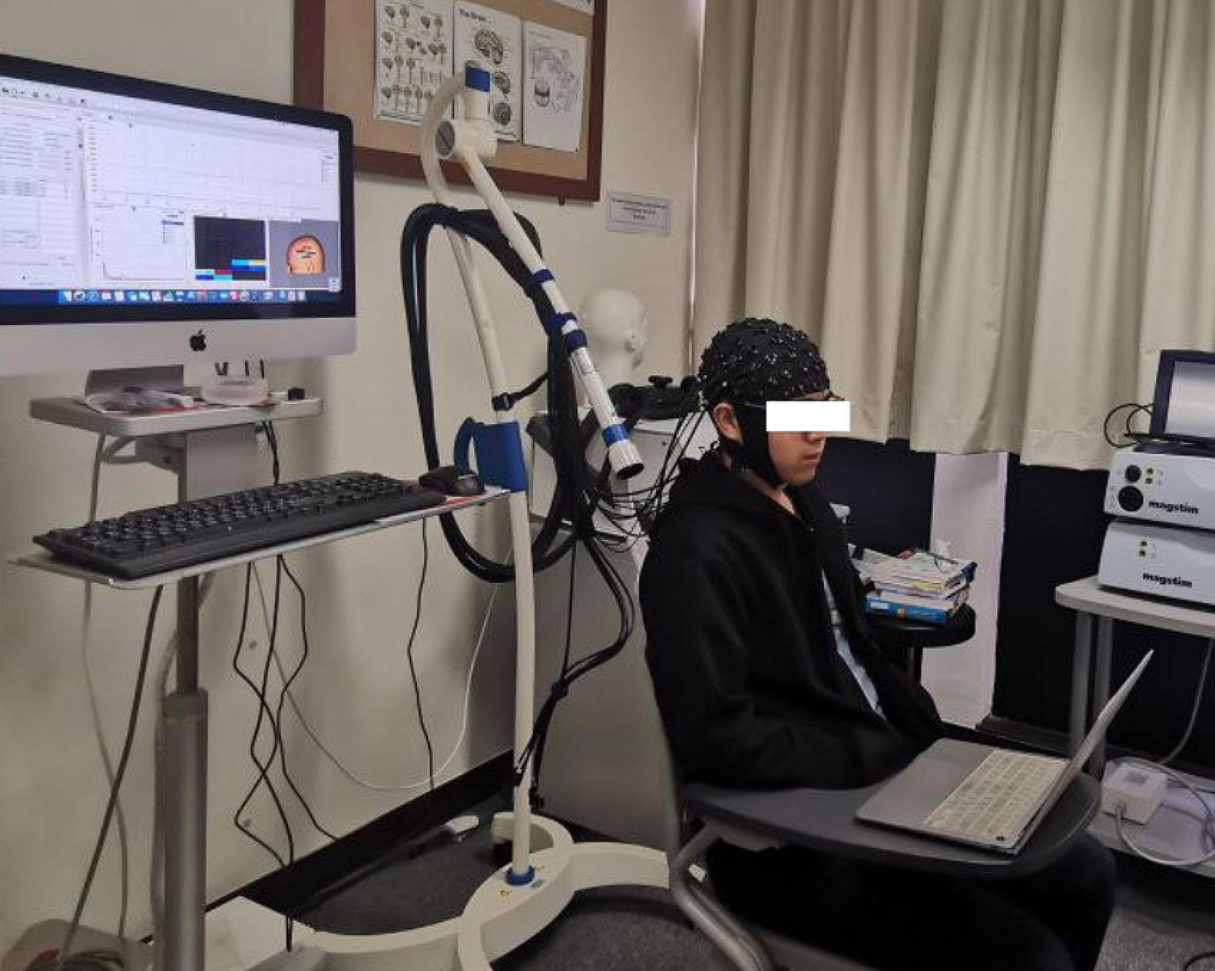
Experimental set-up

**Fig. 2:**
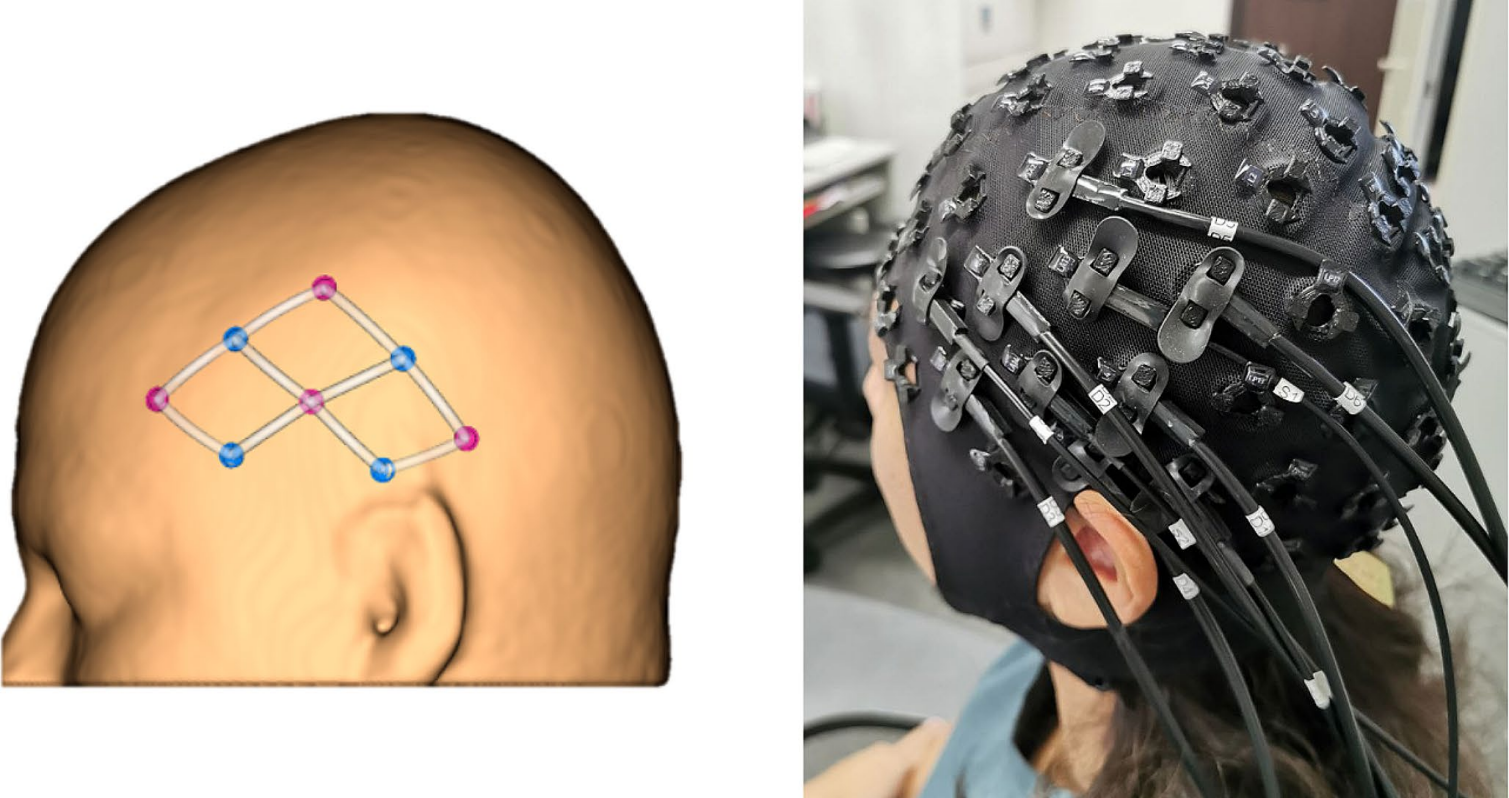
Placement of source (red) and detector (blue) optodes over the left hemisphere

**Fig. 3:**
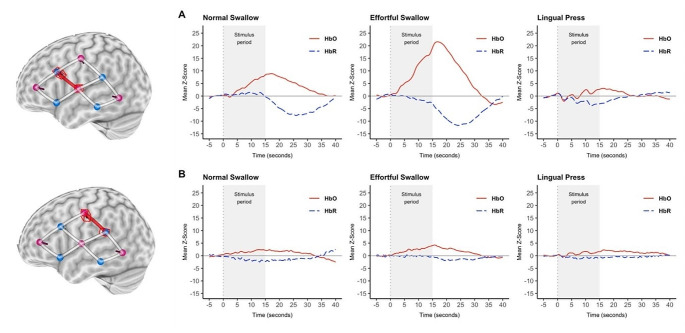
Time plot of oxygenated and deoxygenated z-scores in the (**A**) motor region and (**B**) sensory region across all participants (mean) during normal swallowing, effortful swallowing, and lingual pressing

Participants were seated comfortably all throughout the experiment (Fig. 1). A continuous wave fNIRS equipment (Rogue Research, Montreal, Quebec) was used to measure the relative concentration changes of oxygenated hemoglobin (oxy-Hb) and deoxygenated hemoglobin (deoxy-Hb). Due to the limited number of optodes available with our equipment, only data from left hemisphere was collected (Fig. 2). Short separation channels were not available. For this study, two main regions of interest (ROI) (Fig. 3) on the left primary motor and sensory cortex were chosen based on previous swallowing-related fNIRS studies [17, 22–24]. Specifically, the ROIs were in the left mid-inferior portions of the precentral gyrus and postcentral gyrus found to be active during swallowing and not during finger tapping [25]. These regions were selected to address our question of how effortful swallowing and lingual pressing differ from normal swallowing. The two ROIs will be referred to as ‘motor region’ and ‘sensory region’ throughout this paper.

After fitting and placing the appropriate cap on the participant’s head, the location of the optodes on the scalp was confirmed using a neuronavigation system (Brainsight 2.0, Rogue Research, Montreal, Quebec). To ensure good data quality, source and detector pairs were set approximately 30 mm apart [26] and laser light waves of 690 nm and 830 nm were used to record both oxy- and deoxy-Hb. Data was acquired at a sampling rate of 10 Hz [26]. Signals were set to maximum level gains without oversaturation. A trial run collecting resting state data was initially done to ensure that signals being collected in the channels were stable (i.e., rhythmic presence of spike at ~ 1–1.5 Hz representing cardiac signals [27]). Adjustments to ensure good contact between the optodes and scalp were carried out as necessary (i.e., parting of hair).

### Procedures and Stimuli

**Fig. 4:**
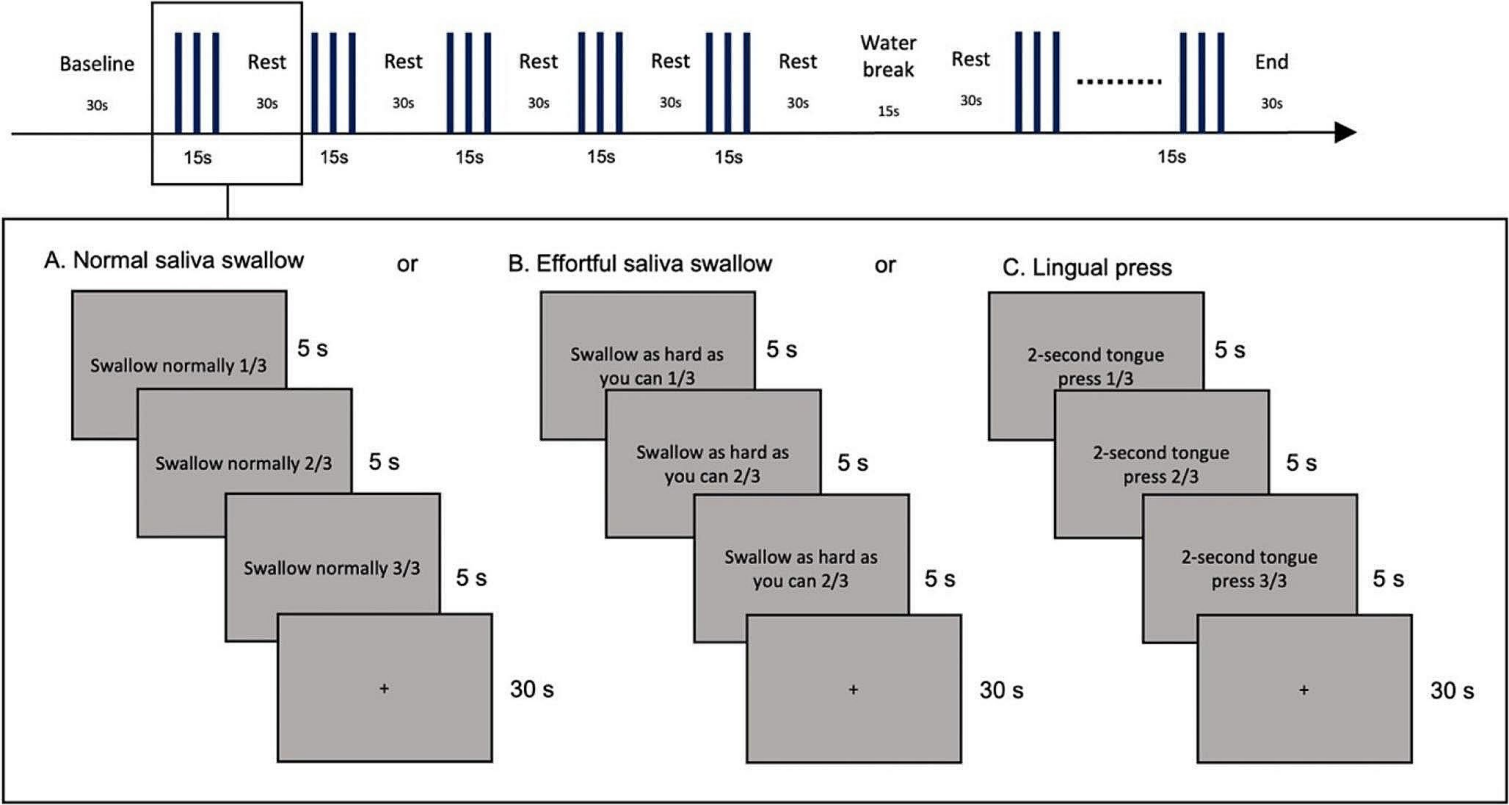
Experimental flow of each block

This study utilized a within-subject block experimental design. All stimuli were presented using PsychoPy [28]. Participants were instructed to keep as still as possible throughout the entire experiment to reduce motion artifacts. They were also reminded not to swallow unless instructed on the screen. The experimental flow is presented in Fig. 4.

Participants were asked to do the following: (1) swallow saliva normally (normal swallow), (2) swallow saliva as hard as possible (effortful swallow), and (3) press tongue as hard as possible against the palate for two seconds (lingual press). Albeit the lack of external biofeedback devices, the lingual press task was selected to represent how tongue-strengthening or lingual resistance exercises are commonly being done in practice [10].

For the effortful swallow, participants were reminded to swallow much harder than the normal swallow. Training was given by a speech-language pathologist prior to data acquisition to ensure that the participants did the tasks correctly. For data collection, each task was performed three times in a single block that lasted 15 s. Five blocks per task was completed (total of 15 experimental blocks). Blocks were randomized and a rest period of 30 s was given between blocks for cortical activation to return to baseline. During the rest period, participants looked at a fixation cross. To moisten the mouth after repeated saliva swallowing, two scheduled water breaks (15 s) wherein participants sipped water via a straw were given after five experimental blocks. The entire experiment lasted for approximately one and a half hours.

### Data Processing

The HomER 3 software was used to analyze the acquired NIRS data. Motion artifact were automatically identified and corrected using spline interpolation. High and low-pass filters of 0.01 and 0.5 Hz respectively were then applied to remove physiological signals (i.e., Mayer waves, respiratory signals, cardiac signals [27]). Visual inspection of hemodynamic response as characterized by a negative correlation between the oxygenated and deoxygenated signals was also done.

Using R, the processed data was exported and converted into z-scores to measure change relative to the baseline (-5 to 0 s; 0 being the start of stimuli). Calculation of z-score was adopted from previous swallowing-related fNIRS studies [17, 20, 29]. This was done for both HbO and HbR values for each participant per condition and ROI (z-score = raw value – mean baseline / SD baseline). For statistical analysis, HbO peak z-scores were also calculated per participant for each condition and ROI. The peak z-score measured the highest level of change, which occurred at around 15 to 20 s after task onset, from the baseline (peak z-score = peak value mean – mean baseline / SD baseline). Time course (-5 to 40 s) values of HbO and HbR z-scores for all participants were also averaged for each condition and ROI (Fig. 3).

### Statistical Methods

Statistical tests were conducted using SPSS. Homogeneity of variances was initially assessed using the Levene’s test (*p* > .05) and a two-way repeated measures analysis of variance (ANOVA) was done taking tasks (normal swallow, effortful swallow, and lingual press) and region of interest (motor and sensory regions) as factors to compare differences in peak z-scores. Post-hoc comparisons between tasks and regions of interest were then performed with Bonferroni correction. A *p*-value of less than 0.05 was considered statistically significant.

## Results

Data from all nine participants with mean age of 26.67 ± 2.74 years old was included for analysis (Table 1). For the purpose of this study, only data from two regions of interest were analyzed. The two-way ANOVA revealed significant main effect of swallowing task [*F*(2, 48) = 10.50, *p* < .001, η_p_^2^ = 0.304] and region of interest [*F*(1, 48) = 21.02, *p* < .001, η_p_^2^ = 0.305] on peak HbO z-score. There was also significant interaction effect between the two [*F*(2, 48) = 7.42, *p* = .002, η_p_^2^ = 0.236]. The mean peak values for each task across the two regions can be found in Table 2.

**Table 1:** Demographic data of participants

	*N*	Age
Male	4	28.5 ± 1.73
Female	5	25.2 ± 2.59
Total	9	26.67 ± 2.74

**Table 2:** Mean peak z-scores for each task across ROIs

	L Motor	L Sensory	Mean difference	*p*-value
Effortful swallow	20.67 ± 11.45	3.48 ± 5.60	17.20	< 0.001*
Normal swallow	8.50 ± 5.26	2.00 ± 5.86	6.50	0.040*
Lingual press	2.74 ± 4.27	2.04 ± 3.47	0.70	0.821

*p < .05

### Across ROIs

Motor region activation was significantly higher than the sensory region during effortful swallowing (*p* < .001) and normal swallowing (*p* = .040) only. No significant difference in activation between the two regions was found during lingual pressing (*p* = .821). The mean difference can be found in Table 2.

### Across Tasks

In the motor region, cortical activation elicited by effortful swallowing was significantly higher than both normal swallowing (*p* < .001) and lingual pressing (*p* < .001) compared to baseline. Activation during normal swallowing and lingual pressing did not differ significantly from each other (*p* = .200). In the sensory region, cortical activity was not statistically different across tasks; however, mean peak z-score was highest during effortful swallowing (*M* = 3.48, *SD* = 5.60), followed by lingual pressing (*M* = 2.04, *SD* = 3.47), and normal swallowing (*M* = 2.00, *SD* = 5.86). The mean difference and Cohen’s d can be found in Table 3.

**Table 3:** Difference in mean peak z-scores across tasks and ROIs

	L Motor	*p*-value	Cohen’d	L Sensory	*p*-value	Cohen’d
Effortful vs. normal	12.17	0.009*	1.37	1.48	1.000	0.26
Normal vs. lingual	5.76	0.200	1.20	− 0.039	1.000	0.01
Effortful vs. lingual	17.93	< 0.001*	2.07	1.437	1.000	0.31

*p < .05

## Discussion

This study aimed to examine brain activity in selected regions of the motor and sensory cortex during normal saliva swallowing, effortful swallowing, and lingual pressing using fNIRS. The type of swallowing task, brain region, and its interaction had a significant effect on the level of cortical activation.

As expected, findings revealed that activation in the motor region was significantly higher than in the sensory region during effortful and normal swallowing. This is in line with previous research [13] and can be explained by the recruitment of various oral and pharyngeal muscles during actual swallowing tasks as compared to lingual pressing. The generation of saliva during both normal and effortful swallowing tasks may have also elicited additional movements in the oral cavity which could have further increased motor activation. Interestingly, activation in the two regions was not statistically different from each other during the lingual pressing task. It is possible that the participants did not push their tongues “hard” enough given the absence of biofeedback and multiple task repetitions (i.e., fatigue); thus, lowering motor activation. The lack of biofeedback may have also increased the need for intrinsic sensory perception (i.e., how hard and how long their tongues were pushing against the palate), thereby increasing sensory activation. A combination of these two factors may have contributed to the comparable activation between the two regions. Follow-up studies should use tools to monitor task execution (i.e., tongue measurement device, sEMG).

Differences in peak z-score in the sensory region were not statistically significant across tasks; however, the mean value during effortful swallowing was relatively the highest, followed by normal swallowing and lingual pressing. Even with ample preparation (i.e., parting of hair to improve scalp-optode contact), we encountered denser hair in the area of the scalp corresponding to the sensory region as compared to the motor region. It is likely that the amplitude of signals obtained from this area was affected, explaining the non-significant results. Further discussion of how to improve data collection using fNIRS is detailed in the Limitations section. Despite this, the mean values concur with previous fMRI findings [13] showing that effortful swallowing elicited higher activation in the sensory region as compared to normal swallowing. On the contrary, our results stating that activation in the sensory region was higher during lingual pressing than normal swallowing differ from Malandraki et al. (2009) [3]. In their study, normal swallowing activated the sensory region more than tongue-tapping. A possible explanation is that the participants in our study were instructed to press their tongues against their palate as hard as possible for two seconds without biofeedback to simulate tongue-strengthening exercises. As compared to tongue-tapping, the lingual pressing task likely requires more neural activity for sensory processing as it relies heavily on sensory information to know whether enough force has been exerted.

### Clinical Implications

This study highlights the potential use of fNIRS in swallowing research. Future studies can further adapt and modify our methodology to explore swallowing-related neurophysiology and plasticity. Understanding the neurological underpinnings of commonly used exercises and techniques may help clinicians establish more targeted dysphagia rehabilitation programs.

The application of fNIRS in rehabilitation as a neurofeedback tool also has strong potential, particularly with patients with acquired dysphagia (e.g., stroke) [30]. In the context of swallowing, Kober and colleagues have examined whether real-time feedback of brain activity could allow participants to voluntary increase or decrease oxy- and deoxy-Hb values through motor imagery [31–33]. Both healthy young and older adults were able to regulate their own brain activity given real-time neurofeedback, and age-related differences were identified [31]. Although promising, more research is needed to confirm whether neurofeedback can be feasibly applied in dysphagia rehabilitation.

### Limitations

Swallowing recruits a wide variety of cortical and subcortical brain regions [1, 3, 4], and differences in hemispheric activation also exist based on the stages (i.e., oral vs. pharyngeal) and tasks (i.e., dry swallow vs. water swallow) at hand [34]. For this study, we only focused on two specific regions of interest in the left hemisphere due to equipment limitations. With fNIRS, activity from subcortical structures such as the insula and thalamus are also not obtained due to poor spatial resolution [27]. The clinical applicability of fNIRS is yet to be established and its use with other neuroimaging techniques (i.e., fMRI, EEG) is strongly recommended to provide a more accurate and comprehensive understanding of brain activity. Moreover, as with other brain imaging studies in the field, the small sample size of this pilot study (*N* = 9) limits the generalizability of the results. Future research should consider replicating the study using robust brain imaging methodologies with a larger number of participants from different populations groups (i.e., older adults, dysphagic patients).

A number of factors could have also affected the fNIRS signals obtained. First, although it was ensured that the cap strap was not fitted too tightly under the participants’ chin, its presence may have altered ‘normal’ task execution in rehabilitation settings. Second, it was inevitable that movements from the suprahyoid muscles during the experimental tasks led to motion artifacts. Motion artifact identification and correction were done during the analysis, however, residual noise was still be observed especially during the lingual pressing task. It is strongly recommended that short-channel separators be used in future fNIRS studies to reduce noise [35]. The use of short-separation channels, paired with proper hair preparation and the use of external accessories to improve and ensure scalp-optode contact throughout the experiment (i.e., bandage wrap), may also improve signal quality particularly in participants with dark and dense hair. Lastly, although training was provided by a certified speech-language pathologist prior to data acquisition, the experimental tasks were not objectively measured or controlled. As a result, force generated during the effortful swallow and lingual pressing tasks may not have been ‘enough’ or equal across participants. The use surface electromyography (sEMG) or tongue pressure measurement devices to ensure that exercise tasks are carried out as expected should be strongly considered.

## Conclusion

This study utilized fNIRS to measure brain activity during normal swallowing, effortful swallowing, and lingual pressing. Activity in selected regions of the precentral motor and postcentral sensory cortices were present across all tasks, but effortful swallowing elicited the highest activation in both regions. Results from this pilot study suggest that fNIRS can be a viable tool to record and distinguish differences in brain activity in the context of swallowing and is a promising neuroimaging tool that can be incorporated in future dysphagia research.

## Data Availability

The data collected from this study are not openly available due to security and sensitivity reasons; however, they are available from the corresponding author upon reasonable request.
